# EZH2 in Cancer Progression and Potential Application in Cancer Therapy: A Friend or Foe?

**DOI:** 10.3390/ijms18061172

**Published:** 2017-05-31

**Authors:** Ke-Sin Yan, Chia-Yuan Lin, Tan-Wei Liao, Cheng-Ming Peng, Shou-Chun Lee, Yi-Jui Liu, Wing P. Chan, Ruey-Hwang Chou

**Affiliations:** 1 Graduate Institute of Biomedical Sciences, China Medical University, No.91, Hsueh-Shih Rd., North Dist., Taichung 40402, Taiwan; n79927@yahoo.com.tw (K.-S.Y.); a8760752@hotmail.com (C.-Y.L.); 2 Center for Molecular Medicine, China Medical University Hospital, No. 2, Yude Rd., North Dist., Taichung 40402, Taiwan; dawn770501@hotmail.com; 3 Da Vinci Minimally Invasive Surgery Center, Chung Shan Medical University Hospital, No. 110, Sec. 1, Chien-Kuo N. Rd., Taichung 40201, Taiwan; jimy5989@gmail.com; 4 Master’s Program of Biomedical Informatics and Biomedical Engineering, Feng Chia University, No. 100, Wenhwa Rd., Seatwen Dist., Taichung 40724, Taiwan; cxz82282@gmail.com (S.-C.L.); erliu@fcu.edu.tw (Y.-J.L.); 5 Department of Automatic Control Engineering, Feng Chia University, No.100, Wenhwa Rd., Seatwen Dist., Taichung 40724, Taiwan; 6 Department of Radiology, Wan Fang Hospital, Taipei Medical University, No. 111, Sec. 3, Singlong Rd., Taipei 11696, Taiwan; wingchan@w.tmu.edu.tw; 7 Department of Biotechnology, Asia University, No. 500, Lioufeng Rd., Wufeng Dist., Taichung 41354, Taiwan

**Keywords:** EZH2, cancer, epigenetic regulation, microRNAs, lncRNAs, EZH2 inhibitors

## Abstract

Enhancer of zeste homolog 2 (EZH2), a histone methyltransferase, catalyzes tri-methylation of histone H3 at Lys 27 (H3K27me3) to regulate gene expression through epigenetic machinery. EZH2 functions as a double-facet molecule in regulation of gene expression via repression or activation mechanisms, depending on the different cellular contexts. EZH2 interacts with both histone and non-histone proteins to modulate diverse physiological functions including cancer progression and malignancy. In this review article, we focused on the updated information regarding microRNAs (miRNAs) and long non coding RNAs (lncRNAs) in regulation of EZH2, the oncogenic and tumor suppressive roles of EZH2 in cancer progression and malignancy, as well as current pre-clinical and clinical trials of EZH2 inhibitors.

## 1. Introduction

Polycomb group (PcG) proteins in mammals play important roles in cell growth and differentiation by regulating expression of downstream genes [1]. PcG proteins contain two core complexes, including polycomb repressive complex 1 and 2 (PRC1 and PRC2). PRC1 has been known to mono-ubiquitinate the histone H2A at Lys 119 by RING1A and RING1B ubiquitin ligases. PRC2 has been considered to catalyze the mono-, di-, and tri-methylation of histone H3 at Lys 27.

Enhancer of zeste homolog 2 (EZH2), a histone methyltransferase served as a catalytic subunit of PRC2, has been known to catalyze tri-methylation of histone H3 at Lys 27 (H3K27me3) by its SET (Su(var)3-9, Enhancer-of-zeste and Trithorax) domain in C-terminus, leading to silencing its target genes involved in cell cycle regulation, cell proliferation, cell differentiation, and cancer progression [2]. EZH2 is an evolutionary conserved gene, which has been identified in many species and contains similar structural motifs and domains among different species including *Drosophila*, frog, mouse, and human. The structural motifs of EZH2 in these species contain conserved SANT (switching-defective protein 3 (Swi3), adaptor 2 (Ada2), nuclear receptor corepressor (NCoR), transcription factor (TF) IIIB) and SET domains. The SANT domain of EZH2 responses to its subcellular localization in the nucleus and is important for chromatin remodeling by promoting binding to histone-tail. It not only regulates chromatin–fiber function, but also provides non-histone protein binding site for other cellular functions [3]. The SET domain of EZH2 provides the enzymatic activity of methyltransferase to regulate expression of downstream genes [4]. The homology of SANT domain is 47%, 47% and 46% in frog, mouse, and human, respectively, comparing to that in *Drosophila*, and SET domain is highly conserved by 93% homology in all species [5].

Mutations and high expression of *EZH2* have been observed in a variety of cancer malignancies, and is correlative with the poor prognosis in different human cancers, indicating the involvement of EZH2 in the development and progression of cancers. Several studies demonstrated that *EZH2* expression can be down-regulated by a variety of microRNAs (miRNAs), which are a class of small non coding RNA, via post-transcriptional gene silencing. In addition, EZH2 protein can be guided by the long non-coding RNAs (lncRNAs) to its target genes via protein-lncRNAs interaction. Due to the potential roles of EZH2 in cancer progression and malignancy, EZH2 has been considered as a relevant therapeutic target for cancers. Accumulated studies indicated that inhibition of EZH2 by the small molecular inhibitors or gene knockdown results in reducing cancer cell growth and tumor formation.

In this review article, we summarized and updated the researches related to miRNAs and lncRNAs in regulation of EZH2, oncogenic and tumor suppressive roles of EZH2 in cancer progression, as well as current pre-clinical and clinical trials of EZH2 inhibitors in cancer therapy.

## 2. Physiological Functions of EZH2

EZH2-mediated histone H3K27me3 in the nucleus is important for PcG proteins to silence chromatin. It has been known that EZH2 participates in embryonic development through regulation of homeobox (Hox) genes. Mutations of PcG genes lead to physical defects in *Drosophila* [6]. Moreover, EZH2 also functions in the cytosol to methylate non-histone proteins. For example, cytosolic EZH2 controls actin polymerization and its related processes, including antigen receptor signaling in T cells and circular dorsal ruffle formation in fibroblasts [7]. EZH2 is involved in regulation of cell division [8], chromatin remodeling [9], DNA replication [10], cell cycle progression [11], and cell senescence [12]. EZH2 also contributes to maintain the properties of pluripotency, self-renewal, proliferation, and regulate differentiation in human embryonic stem cells (ESCs) [13,14]. In mice, depletion of EZH2 causes embryonic death because of anemia caused by the insufficient expansion of hematopoietic stem cells (HSCs) and defective erythropoiesis in fetal liver [15]. In addition, EZH2 not only controls proliferation of plasmablasts and cycling B and T lymphocytes [16,17], but also regulates early B and T cell development [18]. Other studies have shown that EZH2 is required for mammal circadian rhythm [19].

In addition to histone H3K27me3-mediated epigenetic gene silence, EZH2 methylates non-histone proteins, such as GATA-binding protein 4 (GATA4) at Lys 299 for repression of its transcriptional activity [20]. EZH2 also generates a methyl degron on RAR-related orphan receptor alpha (RORα) to regulate its protein stability via methylation-dependent ubiquitination machinery [21]. In contrast, two N-terminal domains of EZH2 interacts directly with β-catenin and estrogen receptor alpha (ERα), and thus links the Wnt and estrogen signaling pathways, leading to gene transactivation and cell cycle progression in breast cancer cells [22]. EZH2 has been shown to interact with PCNA-associated factor (PAF) to the β-catenin complex, and thereby promoting transcriptional activation of Wnt target genes, which is independent of methyltransferase activity of EZH2, in colon cancer cells [23]. In addition, AKT phosphorylates EZH2 at Ser 21. The phosphorylated EZH2 act as a co-activator for critical transcription factors, such as androgen receptor (AR) in prostate cancer cells [24], signal transducer and activator of transcription 3 (STAT3) in glioblastoma stem-like cells [25], and RelA/RelB in estrogen receptors (ER)-negative basal-like breast cancer cells [26], to promote the expression of the target genes of AR, STAT3, and nuclear factor-kappa B (NF-κB), respectively. Therefore, EZH2 functions as a double-facet molecule in regulation of gene expression via repression or activation mechanisms, depending on the different cellular contexts. The targets of EZH2 protein and their roles in regulation of gene expression are listed in Table 1.

## 3. Molecular Regulations of EZH2

### 3.1. MicroRNAs

MicroRNAs (miRNAs), endogenous small non‑coding RNAs, consist of 21–25 nucleotides, which regulate many physiological effects, including fat metabolism, cell proliferation, and cell death [27]. Several miRNAs have been demonstrated to modulate the level of EZH2 through post-transcriptional mechanisms, in which these specific miRNAs are able to bound to EZH2 RNA transcript and regulate its stability, integrity, as well as translation, leading to directly affecting the protein expression of EZH2 [28]. Negative-modulation of these miRNAs promotes EZH2 level and may have implications in cancer progression. Some reports have demonstrated that repression of EZH2 level by miRNAs results in inhibition of cancer metastasis. For example, miR-101 decreases the abilities of invasion and migration in several tumor types containing osteosarcoma in vitro [29], gastric cancer [30], prostate cancer [31], and glioblastoma [32] in vitro and in vivo, through post-transcriptional down-regulation of *EZH2*. Moreover, miR-26a [33,34], miR-138 [35,36,37], miR-124 [38,39,40], miR-98 [41,42], miR-214 [42], miR-30d [43], miR-298 [44], and miR-340 [45] also participate in the post-transcriptional regulation of *EZH2* in different kinds of cancer cells. For example, miR-26a inhibits epithelial–mesenchymal transition (EMT) function and up-regulates tumor suppressor genes, DAB2IP and RUNX3, through post-transcriptional repression of *EZH2* in human hepatocellular carcinoma and lung carcinoma cells in vitro [33]. In addition to the function of miRNAs as tumor suppressors against *EZH2*; however, some miRNAs co-expressed with *EZH2* activates oncogenic pathways. The studies by Bao et al. showed that hypoxia-inducible factor (HIF)-induced co-expression of miR-21, miR-210, and *EZH2* promote aggressiveness of cancer prostate in vitro [46] and pancreatic cancer cells in vitro and in vivo [47] under the hypoxic condition. The above miRNAs related to *EZH2* and their effects on cancer progression are listed in Table 2.

### 3.2. Long Non Coding RNAs

In addition to miRNAs, the long non-coding RNAs (lncRNAs) play important roles in epigenetic regulation. The lengths of lncRNAs are more than 200 nucleotides and their functions are responsible for chromatin modification, transcriptional regulation and post-transcriptional regulation [48]. Emerging evidences indicated that lncRNAs also regulate the functions of EZH2, and their effects were summarized in Table 3. For instance, the Hox transcript antisense RNA (HOTAIR) located in the homeobox C (HOXC) locus interacts with EZH2 protein of PRC2 and lysine specific demethylase 1 (LSD1) complexes. HOTAIR functions as a bridge, which binds to PRC2 with its 5′ domain and LSD1 with its 3′ domain to regulate histone H3K27 tri-methylation and H3K4 de-methylation on repressive chromatin, respectively. Thence, HOTAIR guides these chromatin modifiers to affect the expression of multiple genes involved in a variety of cellular functions [49]. Dysregulation of HOTAIR causes alterations of epigenetic modifications, and thereby promoting cancer progression and malignancy [50]. Metastasis associated lung adenocarcinoma transcript 1 (MALAT-1) locates in the nuclear speckles of mammalian cells, and it takes part in regulating differentiation and proliferation of hematopoiesis [51]. However, MALAT1 can bind to EZH2 and then induces cancer malignant development in aggressive renal cell carcinoma cells [52], esophageal squamous cell carcinoma cells [53] and gastric cancer cells [54] in vitro and in tumor tissue samples. Wang’s study suggested that MALAT1 over-expression increases the proliferation of mantle cell lymphoma (MCL) cells by activating EZH2 and inhibiting p21 and p27 genes in vitro [55]. LncRNA LINC00628 mainly in the nucleus interacts with EZH2 to modulate H3K27me3 level on cell cycle related genes, leading to suppression of proliferation and colony formation of gastric cancer cells in vitro, and tumor size in mouse xenograft models, and thus functions as cancer suppressor in gastric cancer [56]. It also inhibits growth and metastasis via regulation of Bcl-2/Bax/Caspase-3 signal pathway in breast cancer cells in vitro and in tumor tissue samples [57]. Additionally, gastric cancer progression is associated with the lncRNA LINC00673 through the interaction with LSD1 and EZH2, leading to inhibition of KLF2 and LATS2 expression to exert oncogenic functions in vitro and in vivo [58]. LncRNA HOXA11-AS associates with PRC2, LSD1, and DNMT1 to promote cell proliferation, cell cycle progression, and metastasis in gastric cancer [59,60]. LncRNA LINC00511 functions as a scaffold associated with EZH2/PRC2 complexes and regulates their localization to suppress expression of p57 in non-small-cell lung cancer (NSCLC) cells [61]. Similarly, lncRNA LINC00152 and lncRNA CCAT2 interact with EZH2 and guides it to suppress gene expression of p15 and p21 [62] and E-cadherin and LATS2 in gastric cancer cells [63] to promote cancer proliferation in vitro and in vivo. Moreover, lncRNA H19 is important for embryonic development in mammal [64]. The work by Luo et al. indicated that the up-regulation of lncRNA H19 enhances bladder cancer metastasis through the association with EZH2 to activate Wnt/β-catenin and subsequently inhibiting E-cadherin level in vitro and in vivo [65]. In contrast, the lncRNA, which attenuates the functions of EZH2 has also been identified. For example, lncRNA ANCR functions as a scaffold to modulate protein-protein interactions and regulate cell growth, differentiation and metastasis. In the colorectal and breast cancer models, the lncRNA ANCR suppresses invasion and migration properties by down-regulation of EZH2 via ANCR-mediated CDK1-EZH2 interaction and phosphorylation at Thr 345 and Thr 487 on EZH2, facilitating its ubiquitination and degradation [66,67]. The lncRNAs associated with EZH2 and their effects on cancer progression are listed in Table 3.

## 4. Tumor Suppressive Roles of EZH2 in Cancer Progression

Although EZH2 play oncogenic roles in many cancer types, suppression of EZH2 promotes cancer progression in some cancer types, suggesting the tumor suppressive roles of EZH2. The T-cell 1acute lymphoblastic leukemia (T-ALL) is mainly driven by oncogenic activation of NOTCH1 signaling. Activation of NOTCH1 decreases the activity of PRC2 and the histone H3K27me3 repressive mark in T-ALL cells. The study revealed that loss of function of EZH2 by NOTCH1 activation promotes cancer progression of T-ALL [72]. Loss of PRC2-mediated histone H3K27me3 activates hypoxia-inducible transcription factors (HIF)-driven expression of chemokine (C-X-C motif) receptor 4 (CXCR4), leading to metastasis of clear cell renal carcinoma (ccRCC) cells [73]. Moreover, inactivation of EZH2 by phosphorylation at its Ser 21 results in induction of anti-apoptotic genes, IGF1, BCL2, and HIF1A, and increases cell adhesion-mediated drug resistance in multiple myeloma cells [74]. Loss of EZH2 function impairs pancreatic regeneration and facilitates K-Ras (G12D)-driven neoplastic progression in pancreas in vivo, suggesting that EZH2 restrict cancer progression via homeostatic control of pancreatic regeneration [75].

## 5. Oncogenic Roles of EZH2 in Cancer Progression

Although some studies have shown that EZH2 could inhibit cancer progression, the oncogenic roles of EZH2 in tumor progression, malignancy, and poor prognosis still constitute to be mainly accumulated. The amount of EZH2 has been shown to be higher in colorectal cancer (CRC) tumor tissues comparing to that in paired normal tissue. Inhibition of EZH2 by gene knockdown or its inhibitor, DZNep, induces autophagy and apoptosis in CRC cells in vitro [76]. Furthermore, EZH2 could increase cancer proliferation and metastasis in many cancer types, including CRC [77], melanoma [78], oral squamous cell carcinoma (OSCC) [79], and breast cancer [80]. HIF-1α switches the contradictory roles of EZH2/PRC2 via hypoxia state. In HIF-1α inactive state, PRC2 inhibits expression of matrix metalloproteinase genes (MMPs) to suppress invasion. Upon hypoxia, active HIF-1α results in inactivation of PRC2 and release of EZH2, leading to functional switch to EZH2/Forkhead box M1 (FoxM1) complex-induced expression of MMPs and invasion in triple-negative breast cancer. The results demonstrate a oncogenic function of EZH2 independent of PRC2 [80].

EZH2 is also involved in regulation of cell cycle progression and dysregulation of EZH2 accelerates cell proliferation, resulting in cancer development. Knockdown of EZH2 in cholangiocarcinoma cells increases apoptosis and arrests cells in the G1 phase in accordance with elevated levels of p16 and p21 [81]. Suppression of EZH2 by use of EZH2 short hairpin RNA up-regulates the pro-apoptotic proteins, Puma and Bad, and enhances p21 protein expression in both non-small-cell and small-cell lung cancers in vitro [82]. Additionally, EZH2 facilitates tumorigenesis through promoting angiogenesis. Inhibition of EZH2 function by small interfering RNA causes reduction of vascular endothelial growth factor (VEGF) level and cell proliferation, as well as induction of apoptosis in 786-O ccRCC cells. On the contrary, EZH2 over-expression reverses these effects on VEGF level, cell proliferation, and apoptosis in vitro and in vivo [83].

EHZ2 is related to drug resistance and is overexpressed in drug-resistant cancer cell lines. Zhou et al. demonstrated that the protein and mRNA expression of EZH2 is markedly increased in human cisplatin-resistant NSCLC and gastric cancer cells. Silencing EZH2 improves drug resistance to cisplatin, arrests cell cycle in the G0/G1 phase, induces caspase 3/8 activation, as well as up-regulates p15, p21, and p27 expression in vitro [84]. Nuclear accumulation of EZH2 has been demonstrated in pancreatic adenocarcinomas, especially in poorly differentiated one. Genetic depletion of EZH2 sensitizes pancreatic cancer cells to doxorubicin and gemcitabine, leading to induction of apoptosis. In addition, depletion of EZH2 induces expression of p27 and decreases cell proliferation in pancreatic cancer cells [85]. Another report also indicated that silencing EZH2 sensitizes glioblastoma cells to chemotherapeutic drug, TMZ, leading to significant induction of apoptosis and G1/S phase arrest in vitro [86]. According to these studies, the oncogenic and tumor suppressive roles of EZH2 in cancer progression depend on its associated targets, post-translational modifications such as phosphorylation and methylation, and the cancer microenvironment such as hypoxia status. The roles of EZH2 in cancer progression are summarized in Table 4.

Mutations of EZH2 have been identified in many cancer types. We surveyed the cancer genomic data of EZH2 in The Cancer Genome Atlas (TCGA) data base by using the cBioPortal platform [87,88]. The results showed that a variety of genetic alterations including missense mutation, nonsense mutation, frameshift deletion (FS del), and frameshift insertion (FS ins) occur in many cancer types (Table 5). It is well-known that the SET domain in C-terminus of EZH2 is the enzymatic domain for methyltransferase activity and the other region in N-terminus may contribute to the regulatory function of EZH2 (non-SET region) [3,4]. To further predict the potential functional impact of these mutations on EZH2 protein, the predicted functional impact score (FIS) was evaluated. The FIS is classified into four categories, in which neutral and low FIS indicate predicted non-functional protein, whereas medium and high FIS indicate predicted functional protein [89]. Mutations on the SET domain seem to probably have lower FIS, whereas mutations on the non-SET region probably obtain higher FIS, but not all. The characteristics of EZH2 mutations in different cancer types are summarized in Table 5.

## 6. Current Development and Trials of EZH2 Inhibitors

EZH2 regulates expression of the downstream genes, and then affects several physiological functions, including cancer progression, malignancy, and drugs resistance. Therefore, EZH2 is a potential target for cancer therapy and a variety of inhibitors have been developed and their effects on cancers are going to be widely examined. The principles of EZH2 inhibitors and their pre-clinical and clinical trials were described below.

### 6.1. Pre-Clinical Studies

Overexpression EZH2 is important for cancer progression in several cancer types. Accumulated EZH2 inhibitors have been pre-clinically tested, including 3-deazaneplanocin A (DZNep), GSK926, GSK-343, EPZ-005687, EPZ-011989, EI1, UNC-1999 and CPI-169 etc. DZNep is frequently used in many anti-tumor studies, it binds to S-adenosyl-l-homocysteine (SAH) hydrolase inhibitor as a competitive inhibitor of EZH2 to suppress cells angiogenesis and invasion in brain and prostate cancers [133], and to decrease viability in the putative cancer stem cells of biliary tract cancer (BTC) [134,135]. Other PRC inhibitors, such as PTC209 against BMI1 of PRC1, also reduce cell growth in BTC cells [136]. GSK926 [137] and GSK-343 [138] inhibit EZH2 activity to suppress histone H3K27me3 level in breast and prostate cancer cells. In addition, EPZ-005687 and EPZ-011989 decrease histone H3K27me3 mark and kills lymphoma cells with heterozygous mutant EZH2 (Tyr 641 and Ala 677) [139] and inhibits tumor growth of human B cell lymphoma cells [140]. Moreover, in Tyr 641 mutations diffused large B-cell lymphomas cells, EI1 inhibits cell proliferation and induces apoptosis; UNC-1999 inhibits methyltransferase activity of both EZH1 and EZH2 [141,142]. CPI-169 represses EZH2 activity by reducing histone H3K27me3 to suppress cancer progression in germinal center B-cell-like diffuse large B cell lymphoma (GCB-DLBCL) [143]. In addition to the competitive inhibitors of EZH2, a novel strategy to suppress EZH2 by protein degradation has been developed. They recently demonstrated that a gambogenic acid (GNA) derivative, GNA022, covalently binds to Cys 668 at the SET domain of EZH2 and subsequently triggers COOH terminus of Hsp70-interacting protein (CHIP)-mediated ubiquitination of EZH2, leading to promoting EZH2 degradation and inhibiting tumor growth [144]. The aforementioned inhibitors related to EZH2 are performed in the pre-clinical studies in cells or animals and need to be further evaluated in the clinical trials.

### 6.2. Clinical Trials

The *S*-adenosyl-methionine (SAM)-competitive inhibitors of EZH2, including tazemetostat (EPZ-6438, E7438) [145], (*R*)-*N*-((4-Methoxy-6-methyl-2-oxo-1,2-dihydropyridin-3-yl)methyl)-2-methyl-1-(1-(1-(2,2,2-trifluoroethyl)piperidin-4-yl)ethyl)-^1^*H*-indole-3-carboxamide (CPI-1205) [146], GSK2816126, are effective and selective small molecular against EZH2. Tazemetostat, CPI-1205, and GSK2816126, are currently performed in the clinical trials in different cancer types, including lymphomas, kidney tumors, synovial sarcoma, epitheliod sarcoma, mesothelioma, advanced solid tumors, and ovarian cancer. The detail statuses of clinical trials can be surveyed from the database of ClinicalTrials (available online: https://clinicaltrials.gov/). The pre-clinical and clinical trials of drugs against EZH2 were summarized in Table 6.

## 7. Discussion and Conclusions

EZH2 plays important roles in embryonic development, lymphocytes and hematopoietic cell growth, as well as cancer development. A lot of small molecules have been developed as EZH2 inhibitors and their effects on cancers have been examined in vitro and in vivo. The clinical trials of selected EZH2 inhibitors are currently on-going in several types of cancer patients (Table 6). Most EZH2 inhibitors were designed as competitive inhibitors against SAM, the methyl donor of methyltransferases including EZH2. These inhibitors prevent SAM-dependent methyltransferase activity of EZH2 and thus inhibit its enzymatic functions [147]. Besides, a novel strategy to inhibit EZH2 by ubiquitination-mediated degradation has been developed, such as GNA022 [144]. Antibodies against specific protein have been developed, such as Herceptin (trastuzumab) against HER2, a receptor tyrosine kinase (RTK) in the clinical target therapy of cancer patients [148]. However, EZH2 is mainly located in the nucleus and it is difficult for antibodies to penetrate cell membrane into the nucleus and target to EZH2. Thus, antibody-based strategy may not benefit to target nuclear protein. Antisense or RNA interference (RNAi) strategy is commonly used to inhibit specific gene expression in vitro. The main restrictions of antisense- or RNAi-based strategy in clinical trials are delivery system, stability in circulation, and health risk in cancer patients.

Recent study showed that the short-term inhibition of EZH2 can repress tumor cells growth, but long-term inhibition of EZH2 promotes cells proliferation, tumor progression, and increasing DNA damage repair in the xenograft mouse model of glioblastoma [149]. Therefore, the dosage and treatment time of EZH2 inhibitor for cancer therapy should be carefully considered. Moreover, not only oncogenic role but also tumor suppressive role of EZH2 has been demonstrated through distinct mechanisms in different cancer types and conditions (Table 4). Thus, the cancer types need to be selected, and molecular determination of EZH2 status and specific EZH2-associated biomarkers should be performed before targeting EZH2 for cancer therapy.

**Figure 1:**
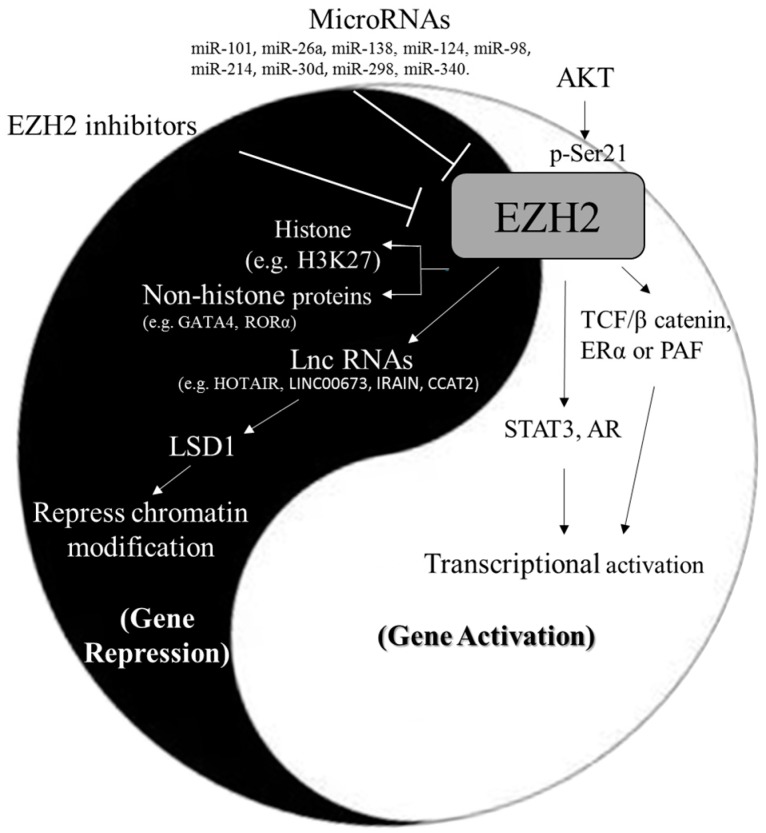
The molecular mechanisms of dual functions of EZH2 in regulation of gene expression. The expression and activity of EZH2 are modulated by miRNAs, lncRNAs, or inhibitors. EZH2 methylates both histone (e.g., H3K27) and non-histone proteins (e.g., GATA4 and RORα) to repress gene expression. In contrast, EZH2 also interacts with transcription fators/co-factors (e.g., STAT3, β-catenin, AR, and ERα) to activate gene expression.

In conclusion, miRNAs (Table 2) and lncRNAs (Table 3) regulate expression and protein functions of EZH2 via post-transcriptional mechanisms or RNA-protein interactions to regulate expression of its targeted genes. EZH2 functions as a double-facet molecule in regulation of gene expression via repression or activation mechanisms, depending on the different cellular contexts (Table 1 and Figure 1). Both oncogenic and tumor suppressive effects of EZH2 (Table 4) have been demonstrated in different cancer types according to distinct molecular actions of EZH2. Furthermore, genetic alterations including missense mutation, nonsense mutation, frameshift deletion, and frameshift insertion occur in many cancer types (Table 5), which may contribute to cancer progression. Therefore, targeting EZH2 is potential therapeutic strategy in cancers (Table 6).

## Figures and Tables

**Table 1:** EZH2 targets and their roles in gene expression.

Target	Subcellular Location	Effects of EZH2	Roles in Gene Expression	Reference
histone H3	nucleus	tri-methylation of histone H3 at Lys 27 (H3K27me3)	silence	[2]
GATA4	nucleus	methylation of GATA4 to inactivate its functions	silence	[20]
RORα	nucleus	methylation-dependent ubiquitination of RORα for its degradation	silence	[21]
ERα/ β-catenin	cytoplasm/nucleus	interaction with β-catenin and ERα to link Wnt and estrogen signaling pathways	activation	[22]
PAF	nucleus	interaction with PAF to the β-catenin complex to activate Wnt target genes	activation	[23]
AKT	cytoplasm/nucleus	phosphorylation of EZH2 at Ser 21 to activate its function	activation	[24]
RelA/RelB	cytoplasm/nucleus	interaction with RelA/RelB to activate NF-κB	activation	[26]
STAT3	cytoplasm/nucleus	co-activator of STAT3	activation	[25]
AR	cytoplasm/nucleus	co-activator of AR	activation	[24]

**Table 2:** The microRNAs related to *EZH2* and their functions.

MicroRNAs	Effect	Roles in Cancer	Reference
miR-101	down-regulation of *EZH2* expression to inhibit cell proliferation, invasion, and migration abilities in osteosarcoma cells (F5M2) in vitro, gastric cancer cells (MKN-45) in vitro and in vivo (xenograft), prostate cancer cells (SKBr3 and DU145) in vitro and in vivo (xenograft), glioblastoma cells (U87, U118, U251, and U373) and rat GBM cells (C6) in vitro and in vivo (xenograft)	suppressor	[29,30,31,32]
miR-26a	inhibition of *EZH2* to suppress EMT in human hepatocellular carcinoma; up-regulation of tumor suppressor genes (e.g., DAB2IP and RUNX3) to inhibit cell growth and metastasis in lung carcinoma cells (A549) in vitro and osteosarcoma cells (MG-63 and U20S) in vitro and in tumor tissue samples (in situ)	suppressor	[33,34]
miR-138	inhibition of *EZH2* to suppress tumor growth and EMT in NSCLC cells (A549, SPC-A1, SK-MES-1, and H460) and normal human bronchial epithelial cells (16HBE) in vitro and in vivo and in tumor tissue samples (in situ) and squamous cell carcinoma cells (1386Ln and 686Tu) in vitro; targeting *EZH2* to induce senescence in human clear cell renal cell carcinoma cells (SN-12) in vitro and in vivo and in tumor tissue samples (in situ)	suppressor	[35,36,37]
miR-124	targeting *EZH2* to suppress proliferation in gastric cancer cells (MKN-45, MGC-803, SGC-7901 and AGS) in vitro and in vivo and in tumor tissue samples (in situ); inhibit *ROCK2* and *EZH2* to repress invasiveness and metastasis in hepatocellular carcinoma cells (Hep3B, Bel-7402, SMMC-7721 and MHCC-LM9) in vitro and in vivo and in tumor tissue samples (in situ)	suppressor	[39,40]
miR-98	inhibition of *EZH2* to suppress cells migration and invasion in human esophageal squamous cell carcinoma cells (Eca109) in vitro and in tumor tissue samples (in situ); inhibit cells proliferation via targeting *EZH2* to regulate Wnt/β-catenin signaling pathway in hepatocellular carcinoma cells (HepG2) in vitro and in tumor tissue samples (in situ)	suppressor	[41,42]
miR-214	inhibition of *EZH2* to suppress migration and invasion in human esophageal squamous cell carcinoma cells (Eca109) in vitro and in tumor tissue samples (in situ)	suppressor	[42]
miR-30d	targeting *EZH2* to inhibit migration and invasion in human esophageal squamous cell carcinoma cells (ECA109 and KYSE410) in vitro and in tumor tissue samples (in situ)	suppressor	[43]
miR-298	reduction of *EZH2* expression to suppress migration and invasion in epithelial ovarian cancer cells (SKOV3 and OVCAR3) in vitro and in tumor tissue samples (in situ)	suppressor	[44]
miR-340	targeting *EZH2* to inhibit cancer progression in squamous cell carcinoma cells (Hep-2) in vitro and in tumor tissue samples (in situ)	suppressor	[45]
miR-21	hypoxic state, co-expression with *EZH2*, *IL6*, *HIF-1α*, and *VEGF* in pancreatic cancer cells (AsPC-1 and MiaPaCa-2) in vitro and in vivo and prostate cancer cells (PC-3 and LNCaP) in vitro	pro-oncogenic	[46,47]
miR-210	hypoxic state, co-expression with *EZH2*, *IL6*, *HIF-1α*, and *VEGF* in pancreatic cancer cells (AsPC-1 and MiaPaCa-2) in vitro and in vivo and prostate cancer cells (PC-3 and LNCaP) in vitro	pro-oncogenic	[46,47]

**Table 3:** The lncRNAs associated with EZH2 and their functions.

lncRNAs	Role	Function	Reference
HOTAIR	interaction with EZH2/PRC2 and LSD1 as a repressive chromatin modifier	promoting cancer metastasis via re-localization remodeling of chromatin by PRC2 in many cancer types, including esophageal squamous cell carcinoma cells (KYSE30) in vitro and in tumor tissue samples (in situ), breast cancer cells (MDA-MB-231) in vitro and in vivo (xenograft), and in tumor tissue samples (in situ), and epithelial ovarian cancer cells (SKOV3.ip1 , HO8910-PM, and HEY-A8) in vitro and in vivo (xenograft), and in tumor tissue samples (in situ)	[49,50]
MALAT-1	association with EZH2	activating EZH2 to suppress p21 and p27 expression and promote cell proliferation in MCL cells (Mino and Jeko-1) in vitro; binding to EZH2 to regulate cancer malignant development in many cancer types, including renal cell carcinoma cells (A-498 and 786-O) in vitro and in tumor tissue samples (in situ), esophageal squamous cell carcinoma cells (TE7) in vitro and in tumor tissue samples (in situ), gastric cancer cells (MKN45 and AGS) in vitro and in tumor tissue samples (in situ), and in MCL cells (Mino and Jeko-1) in vitro and in tumor tissue samples (in situ)	[51,52,53,54,55,68,69]
LINC00628	association with EZH2	interacting with EZH2 to reduce expression of cell cycle related genes in gastric cancer cells (SGC7901 and MGC-803) in vitro, and tumor size in vivo (xenograft); inhibiting cancer cells growth and metastasis via regulation of Bcl-2/Bax/Caspase-3 signal pathway in breast cancer cells (LCC2 and MCF-7) in vitro and tumor tissue samples (in situ)	[56,57]
LINC00673	a scaffold for interaction with LSD1 and EZH2	inhibiting expression of KLF2 and LATS2 via association with EZH2 and LSD1 to exert oncogenic functions in gastric cancer cells (BGC823, SGC7901, MGC803, and AGS) in vitro and in vivo (xenograft)	[58]
HOXA11-AS	a scaffold for association with PRC2, LSD1, and DNMT1	promotes cell proliferation, cell cycle progression and metastasis in gastric cancer cells (BGC823 and AGS cells) in vitro, in vivo (xenograft) and in tumor tissue samples (in situ)	[59,60]
IRAIN	interaction with EZH2 and LSD1	interacting with EZH2 and LSD1 to decrease expression of KLF2 and p15 and inhibit apoptosis and cause cycle arrest in pancreatic cancer cells (AsPC-1, BxPC-3, and Panc-1) in vitro and in tumor tissue samples (in situ)	[70]
LINC00511	a scaffold for interaction with EZH2/PRC2 to regulate their localization and functions	suppressing expression of p57 through the association with EZH2 in NSCLC cells (A549 and SPC-A-1) in vitro*,* in vivo, and in tumor tissue samples (in situ)	[61]
LINC00152	association with EZH2	promoting gastric cancer cells (SGC-7901 and BGC-823) progression through recruiting EZH2 to suppress p15 and p21 or promote EMT in vitro and metastasis in vivo, and in tumor tissue samples (in situ*)*	[62]
CCAT2	association with EZH2 and LSD1	suppressing expression of E-cadherin and LATS2 levels in gastric cancer cells (MKN45 and BGC-823) in vitro and in tumor tissue samples (in situ)	[63]
H19	association with EZH2	association with EZH2 to activate Wnt/β-catenin and downregulate E-cadherin in bladder cancer cells (RT4 and T24) in vitro and in vivo; interaction with miR-630 to regulate EZH2 level in nasopharyngeal carcinoma cells (CNE2 and HONE1) in vitro and in tumor tissue samples (in situ)	[64,65,71]
ANCR	a scaffold for association with EZH2 and CDK1	decreasing EZH2 to inhibit invasion and metastasis in colorectal cancer cells (SW620) in vitro and in vivo (xenograft); recruiting CDK1 and EZH2 to phosphorylate EZH2 at T345 and T487, hence facilitating EZH2 ubiquitination to degradation, leading to attenuation malignancy in breast cancer cells (MDA-MB-231) in vitro and in vivo (xenograft) and in tumor tissue samples (in situ)	[66,67]

**Table 4:** Roles of EZH2 in cancer progression.

Cell type	Model	Function	Roles in Cancer	Reference
T-cell acute lymphoblastic leukemia (T-ALL)	CUTLL1, Loucy, Jurkat, MOLT3, HPB-ALL, P12-ichikawa, DND41, CEM2 cell lines (in vitro and in vivo), and tumor tissue samples (in situ)	Loss of EZH2 functions by NOTCH1 pathway and promotes cancer progression.	suppressive	[72]
Clear cell renal carcinoma (ccRCC)	786-O, RFX-631, and OS-RC-2 cell lines (in vitro and in vivo) and in tumor tissue samples (in situ)	Loss of PRC2-mediated histone H3K27me3 activates HIF-driven CXCR4 and increases tumor invasion (suppressive); Overexpression of EZH2 increases VEGF level and cell proliferation (oncogenic).	suppressive or oncogenic	[73,83]
Pancreatic cells	EZH2 knockout mice (in vivo)	Loss of EZH2 facilitates K-Ras^G12D^-driven tumor formation	suppressive	[75]
Colorectal cancer (CRC) cells	SW620 cell line (in vitro) and in tumor tissue samples (in situ), and RKO and HCT116 cell lines (in vitro)	Inhibition of EZH2 induces autophagy and apoptosis and suppresses cell proliferation and migration.	oncogenic	[76,77]
Melanoma	XB2 and Melan-a cell lines (in vitro and in vivo) and in tumor tissue samples (in situ)	EZH2 represses distinct tumor suppressor genes to promote metastasis.	oncogenic	[78]
Oral squamous cells carcinoma (OSCC)	Tca8113, Tb, and Ts cell lines (in vitro) and in tumor tissue samples (in situ)	Reducing EZH2 inhibits cell proliferation, migration, metastasis, and induces apoptosis.	oncogenic	[79]
Breast cancer	MDA-MB231, HS578T, and BT549 cell lines (in vitro) and in tumor tissue samples (in situ)	PRC2 inhibits expression of MMPs to suppress invasion in normoxia (suppressive). Upon hypoxia, HIF-1α inactivates PRC2 and leads EZH2 to functional switch to EZH2/FoxM1-induced expression of MMPs and invasion (oncogenic).	suppressive or oncogenic	[80]
Cholangiocarcinoma cells	RBE and TFK-1 cell lines (in vitro) and in tumor tissue samples (in situ)	Inhibition of EZH2 induces G1 phase arrest, reduces cells growth, and induce apoptosis.	oncogenic	[81]
Small cell lung cancer (SCLC)	HTB-175, NCI-H526, HTB-171, HTB-119, and NCI-H524 cell lines (in vitro)	Suppression of EZH2 reduces cells in S or G2/M phases and increases p21 expression.	oncogenic	[82]

**Table 5:** The characteristics of EZH2 mutations in different cancer types.

Cancer Type	Mutation Site	Mutation Type ^a^	Location	Predicted Functional Impact Score (FIS) ^b^	Reference
Lung adenocarcinoma	A715V	missense	SET domain	1.19 (low)	[90,91] TCGA data base
	R34L	missense	non-SET region	1.50 (low)	
	A627E	missense	SET domain	1.55 (low)	
	R502Q	missense	non-SET region	2.98 (medium)	
	E346K	missense	non-SET region	1.25 (low)	
	A622E	missense	non-SET region	1.55 (low)	
	R497Q	missense	non-SET region	2.98 (medium)	
	E341K	missense	non-SET region	1.25 (low)	
Lung squamous cell carcinoma	E374Q	missense	non-SET region	2.08 (medium)	[92] TCGA data base
	S551*	nonsense	non-SET region	-	
	Q548E	missense	non-SET region	1.99 (medium)	
	R308L	missense	non-SET region	2.71 (medium)	
	E379Q	missense	non-SET region	2.08 (medium)	
	A345T	missense	non-SET region	0.55 (neutral)	
	S556*	nonsense	non-SET region	-	
	Q553E	missense	non-SET region	1.99 (medium)	
Small cell lung cancer	D185G	missense	non-SET region	1.04 (low)	[93,94]
	S40C	missense	non-SET region	-	
Pan-lung cancer	A715V	missense	SET domain	1.19 (low)	[95]
	A622E	missense	non-SET region	1.55 (low)	
	R497Q	missense	non-SET region	2.98 (medium)	
	R34L	missense	non-SET region	1.50 (low)	
	E341K	missense	non-SET region	1.25 (low)	
	K510R	missense	non-SET region	1.87 (low)	
	E374Q	missense	non-SET region	2.08 (medium)	
	S551*	nonsense	non-SET region	-	
	Q548E	missense	non-SET region	1.99 (medium)	
	G5R	missense	non-SET region	0.90 (low)	
	P262I	missense	non-SET region	-	
	R64M	missense	non-SET region	1.79 (low)	
	D186N	missense	non-SET region	1.50 (low)	
	K39E	missense	non-SET region	1.65 (low)	
	R685G	missense	SET domain	-	
	H613Q	missense	non-SET region	1.43 (low)	
	K505Yfs*3	FS del	non-SET region	-	
	N310S	missense	non-SET region	0.41 (neutral)	
	R27*	nonsense	non-SET region	-	
Breast invasive carcinoma	S644*	nonsense	SET domain	-	[96,97] TCGA data base
	E197Rfs*12	FS del	non-SET region	-	
	T718I	missense	SET domain	0.45 (neutral)	
	S639*	nonsense	SET domain	-	
Metastatic breast cancer	A687V	missense	SET domain	1.14 (low)	[98]
	L315V	missense	non-SET region	-	
Liver hepatocellular carcinoma	C580*	nonsense	non-SET region	-	[99] TCGA data base
	E640*	nonsense	SET domain	-	
	G395Efs*29	FS del	non-SET region	-	
	I689S	missense	SET domain	−1.22 (neutral)	
Pediatric ewing sarcoma	Y646H	missense	SET domain	4.61 (high)	[100]
Ewing sarcoma	A677G	missense	SET domain	2.31 (medium)	[101]
	Y641H	missense	SET domain	4.61 (high)	
	Y641F	missense	SET domain	-	
Clear cell renal cell carcinoma	K6M	missense	non-SET region	−0.46 (neutral)	[102] TCGA data base
	Q540*	nonsense	non-SET region	-	
	D187Gfs*2	FS ins	non-SET region	-	
	Q545*	nonsense	non-SET region	-	
Prostate adenocarcinoma	R16W	missense	non-SET region	1.04 (low)	TCGA data base
Pancreatic adenocarcinoma	R658I	missense	SET domain	2.44 (medium)	TCGA data base
	V582A	missense	non-SET region	1.80 (low)	
	A237S	missense	non-SET region	−0.20 (neutral)	
Merged cohort of lower grade glioma (LGG) and glioblastoma multiforme (GBM)	M121I	missense	non-SET region	2.48 (medium)	[103]
Glioblastoma multiforme (GBM)	E396Kfs*22	FS del	non-SET region	-	[104] TCGA data base
	K510R	missense	non-SET region	1.87 (low)	
	K515R	missense	non-SET region	1.87 (low)	
	M121I	missense	non-SET region	2.48 (medium)	
	E401Kfs*22	FS del	non-SET region	-	
Low-grade glioma (LGG)	G11R	missense	non-SET region	0.69 (neutral)	[105]
Medulloblastoma	H706N	missense	SET domain	-	[106]
Colorectal adenocarcinoma	C663S	missense	SET domain	0.53 (neutral)	[107,108,109] TCGA data base
	R213H	missense	non-SET region	−0.34 (neutral)	
	E720K	missense	SET domain	2.93 (medium)	
	E169D	missense	non-SET region	1.45 (low)	
	E725K	missense	SET domain	2.93 (medium)	
	R216Q	missense	non-SET region	1.04 (low)	
	V13A	missense	non-SET region	-	
	R16Q	missense	non-SET region	-	
	N697D	missense	SET domain	-	
	P577L	missense	non-SET region	-	
	R354H	missense	non-SET region	-	
	L252P	missense	non-SET region	-	
	R347W	missense	non-SET region	-	
	D202Y	missense	non-SET region	-	
	M667T	missense	SET domain	-	
	R566C	missense	non-SET region	-	
	R313W	missense	non-SET region	-	
	S368C	missense	non-SET region	-	
	R25Q	missense	non-SET region		
	I223F	missense	non-SET region	-	
	A255T	missense	non-SET region	-	
	N152Ifs*15	FS del	non-SET region	-	
	R347Q	Missense	non-SET region	-	
	S368N	missense	non-SET region	-	
	D536E	missense	non-SET region	-	
	R353C	missense	non-SET region	2.19 (medium)	
	N423T	missense	non-SET region	0.55 (neutral)	
Bladder urothelial carcinoma	K201E	missense	non-SET region	−0.53 (neutral)	[110,111]
	S271Y	missense	non-SET region	2.65 (medium)	
	F145Y	missense	non-SET region	-	
Bladder cancer	A596T	missense	non-SET region	0.64 (neutral)	[112,113]
	T80Lfs*6	FS del	non-SET region	-	
	A677G	missense	SET domain	2.31 (medium)	
	S639L	missense	SET domain	0.56 (neutral)	
Esophageal squamous cell carcinoma	V621M	missense	non-SET region	2.33 (medium)	[114]
Esophageal adenocarcinoma	E333Q	missense	non-SET region	2.19 (medium)	[115] TCGA data base
	F171S	missense	non-SET region	2.60 (medium)	
	P488S	missense	non-SET region	1.47 (low)	
	D192Y	missense	non-SET region	0.90 (low)	
Esophagogastric cancer	D192Y	missense	non-SET region	0.90 (low)	[116]
Stomach adenocarcinoma	R18C	missense	non-SET region	1.94 (medium)	[117] TCGA data base
	E740K	missense	non-SET region	1.16 (low)	
	M662T	missense	SET domain	0.33 (neutral)	
	S43I	missense	non-SET region	0.90 (low)	
	N668S	missense	SET domain	0.77 (neutral)	
Cervical squamous cell carcinoma and endocervical adenocarcinoma	P364S	missense	non-SET region	1.25 (low)	TCGA data base
	D293H	missense	non-SET region	2.81 (medium)	
	S695L	missense	SET domain	3.46 (medium)	
Head and neck squamous cell carcinoma	R357W	missense	non-SET region	1.10 (low)	[118,119] TCGA data base
	D189N	missense	non-SET region	0.00 (neutral)	
	R362W	missense	non-SET region	1.10 (low)	
	P115S	missense	non-SET region	2.85 (medium)	
	R362Q	missense	non-SET region	−0.29 (neutral)	
	R216W	missense	non-SET region	1.04 (low)	
	I264R	missense	non-SET region	2.62 (medium)	
	Y181C	missense	non-SET region	2.44 (medium)	
	S533L	missense	non-SET region	2.38 (medium)	
Testicular germ cell cancer	K510R	missense	non-SET region	1.87 (low)	TCGA data base
	P115T	missense	non-SET region	2.85 (medium)	
Cholangiocarcinoma	H282N	missense	non-SET region	2.30 (medium)	TCGA data base
Skin cutaneous melanoma	Y641N	missense	SET domain	4.61 (high)	[120,121] TCGA data base
	R342Q	missense	non-SET region	1.15 (low)	
	S229L	missense	non-SET region	1.62 (low)	
	Y641F	missense	SET domain	-	
	P746S	missense	non-SET region	0.00 (neutral)	
	R34P	missense	non-SET region	1.50 (low)	
	Y641S	missense	SET domain	4.61 (high)	
	S533L	missense	non-SET region	2.38 (medium)	
	R216Q	missense	non-SET region	1.04 (low)	
	P132S	missense	non-SET region	2.93 (medium)	
	R355G	missense	non-SET region	2.19 (medium)	
	P426S	missense	non-SET region	1.38 (low)	
	D142V	missense	non-SET region	2.90 (medium)	
	C530W	missense	non-SET region	3.57 (high)	
	G704S	missense	SET domain	2.46 (medium)	
	A226V	missense	non-SET region	2.79 (medium)	
	T4I	missense	non-SET region	1.10 (low)	
	T4P	missense	non-SET region	0.41 (neutral)	
Cutaneous squamous cell carcinoma	R685C	missense	SET domain	4.42 (high)	[122]
	Y641S	missense	SET domain	4.61 (high)	
Desmoplastic melanoma	S84L	missense	non-SET region	0.20 (neutral)	[123]
Hepatocellular carcinomas	N670S	missense	SET domain	-	[124]
Ampullary carcinoma	Q323K	missense	non-SET region	-	[125]
Leukemia	R342Q	missense	non-SET region	1.15 (low)	[126]
Acute myeloid leukemia	E740Afs*24	FS ins	non-SET region	-	[127] TCGA data base
	I739Mfs*25	FS ins	non-SET region	-	
	R685H	missense	SET domain	2.67 (medium)	
	E745Afs*24	FS ins	non-SET region	-	
	I744Mfs*25	FS ins	non-SET region	-	
	R690H	missense	SET domain	2.67 (medium)	
Hypodiploid acute lymphoid leukemia	N670K	missense	SET domain	1.89 (low)	[128]
	R679H	missense	SET domain	2.17 (medium)	
	G159R	missense	non-SET region	2.80 (medium)	
Lymphoid neoplasm diffuse large B-cell lymphoma	Y646F	missense	SET domain	-	TCGA data base
	Y646S	missense	SET domain	high	
	K665R	missense	non-SET region	low	
	K665E	missense	non-SET region	low	
	D185H	missense	non-SET region	low	
Diffuse large B-Cell lymphoma	Y641F	missense	SET domain	-	[129]
	Y641N	missense	SET domain	4.61 (high)	
	A687V	missense	SET domain	1.14 (low)	
Myelodysplasia	K713Efs*12	FS del	SET domain	-	[130]
	D659A	missense	SET domain	2.00 (medium)	
Uterine carcinosarcoma	R608Q	missense	non-SET region	2.63 (medium)	[131] TCGA data base
	E59*	nonsense	non-SET region	-	
Uterine corpus endometrial carcinoma	E740K	missense	non-SET region	1.16 (low)	[132] TCGA data base
	R497Q	missense	non-SET region	2.98 (medium)	
	Y447*	nonsense	non-SET region	-	
	Q540P	missense	non-SET region	2.25 (medium)	
	D233Y	missense	non-SET region	1.95 (medium)	
	E162*	nonsense	non-SET region	-	
	F673C	missense	SET domain	2.69 (medium)	
	R78H	missense	non-SET region	2.14 (medium)	
	E246*	nonsense	non-SET region	-	
	E396*	nonsense	non-SET region	-	
	R349C	missense	non-SET region	0.90 (low)	
	K241Q	missense	non-SET region	2.51 (medium)	
	E721D	missense	SET domain	4.12 (high)	
	R207Q	missense	non-SET region	0.20 (neutral)	

^a^ FS del: frameshift deletions; FS ins: frameshift insertions. ^b^ functional impact score (FIS): neutral and low indicate predicted non-functional; medium and high indicate predicted functional [89]. *: truncating mutations.

**Table 6:** Pre-and clinical trials of drugs related to EZH2.

Drug	Role	Trial	Stage	Reference
3-Deazaneplanocin A (DZNep)	*S*-adenosyl-l-homocysteine (SAH) hydrolase inhibitor	many cancer cell lines, such as prostate cancer, brain cancer, and biliary tract cancer cells (EGI-1)	pre-clinical	[133,134,135]
GSK926	SAM-competitive inhibitors of EZH2	OVCAR10, UPN289 and SKOV3 epithelial ovarian cancer (EOC) cell lines	pre-clinical	[137]
GSK343	SAM-competitive inhibitors of EZH2	HCC1806, Sk-Br-3 and ZR-75-1 breast cancer cells and LNCaP, PC3 and LNcaP prostate cancer cells	pre-clinical	[138]
EPZ-005687	Inhibitor of EZH2 T641 and A677 mutants	mutant lymphoma cells (heterozygous Tyr 641 or Ala 677)	pre-clinical	[139]
EPZ-011989	selective, oral inhibitor of EZH2	EZH2 mutant WSU-DLCL2 (Y641F) and DLBCL cell lines in xenograft mouse model	pre-clinical	[140]
EI1	SAM-competitive inhibitors of EZH2	EZH2 mutat cell lines: WSU-DLCL2 (Y641F), SH-DHL6 (Y641N), and DLBCL cells wild-type EZH2 cell lines: OCI-Y19, GA10, DLBCL, and G401 rhabdoid tumor cells	pre-clinical	[141]
UNC-1999	SAM-competitive inhibitors of EZH2 and EZH1	MCF7 breast cancer cells, EZH2 mutant DB cells (Y641N) and DLBCL cells, and HEK293T human embryonic kidney cells	pre-clinical	[142]
CPI-169	SAM-competitive inhibitors of EZH2	lymphoma cell lines, such as GCB, ABC-DLBCL, BL, and MCL cells	pre-clinical	[143]
GNA022	CHIP-mediated ubiquitination and degradation of EZH2	HN-6 human epithelial cancer cells, A549 lung cancer cells, human head and neck cancer cell lines: UMSCC-12 SCC-25, HN-4, HN-6, HN-12, HN-13, HN-30, Cal-27, KB, and KB/VCR, and breast cancer cell lines: MDA-MB-231 and MDA-MB-468, and SMMC-7721 hepatocyte carcinoma cells	pre-clinical	[144]
Tazemetostat (EPZ-6438, E7438)	SAM-competitive inhibitors of EZH2	10 clinical studies on going in B-cell non-Hodgkin’s lymphoma, diffuse large B-cell lymphoma, B-cell lymphomas, follicular lymphoma, malignant rhabdoid tumors (MRT), rhabdoid tumors of the kidney (RTK), atypical teratoid rhabdoid tumors (ATRT), synovial sarcoma, epitheliod sarcoma, mesothelioma, advanced solid tumors, selected tumors with rhabdoid features, INI1-negative tumors, malignant rhabdoid tumor of ovary, renal medullary carcinoma	Phase II	-
CPI-1205	SAM-competitive inhibitors of EZH2	1 clinical study on going in B-cell lymphomas	Phase I	-
GSK2816126	SAM-competitive inhibitors of EZH2	1 clinical study on going in relapsed/refractory diffuse large B cell lymphoma, transformed follicular lymphoma, other non-Hodgkin’s lymphomas, solid tumors and multiple myeloma	Phase I	-
